# Oxygenation index in the first three weeks of life is a predictor of bronchopulmonary dysplasia grade in very preterm infants

**DOI:** 10.1186/s12887-023-03835-3

**Published:** 2023-01-13

**Authors:** Fu-Sheng Chou, Rebekah M. Leigh, Srinandini S. Rao, Arvind Narang, Hung-Wen Yeh

**Affiliations:** 1 Division of Neonatology, Department of Pediatrics, Loma Linda University School of Medicine, Loma Linda, CA USA; 2 Department of Neonatal-Perinatal Medicine, Kaiser Permanente Riverside Medical Center, 10800 Magnolia Ave., Riverside, CA USA; 3 Loma Linda University School of Medicine, Loma Linda, CA USA; 4 Business Intelligence and Data Governance, Loma Linda University Health, Loma Linda, CA USA; 5 Division of Health Services and Outcomes Research, Children’s Mercy Research Institute, Kansas City, MO USA

**Keywords:** Very preterm infants, Bronchopulmonary dysplasia, Oxygenation index, Generalized additive mixed modeling, Longitudinal analysis

## Abstract

**Background:**

The new bronchopulmonary dysplasia (BPD) grading system was developed based on its correlation with long-term respiratory and neurodevelopmental outcomes and may provide better personalized prognostication. Identifying early-life predictors for accurate BPD grade prediction may allow interventions to be tailored to individual needs. This study aimed to assess whether oxygenation index (OI) dynamics in the first three weeks of life are a predictor of BPD grade.

**Methods:**

A single-center retrospective study was performed. Generalized additive mixed modeling was used to model OI trajectories for each BPD grade subgroup. A multinomial regression model was then developed to quantify the association between OI dynamics and BPD grade.

**Results:**

Two hundred fifty-four infants were identified for inclusion in the trajectory modeling. A total of 6,243 OI data points were available for modeling. OI trajectory estimates showed distinct patterns in the three groups, most prominent during the third week of life. The average daily OI change was -0.33 ± 0.52 (*n* = 85) in the No-BPD group, -0.04 ± 0.75 (*n* = 82) in the Low-Grade BPD group, and 0.22 ± 0.65 (*n* = 75) in the High-Grade BPD group (*p* < 0.001). A multinomial regression analysis showed the initial OI value and the average daily OI change both independently correlated with BPD grade outcomes after adjusting for birth gestation, birth weight z-score, sex, and the duration of invasive ventilation.

**Conclusion:**

Early-life OI dynamics may be a useful independent marker for BPD grade prediction. Prospective studies may be warranted to further validate the findings.

**Supplementary Information:**

The online version contains supplementary material available at 10.1186/s12887-023-03835-3.

## Introduction

Bronchopulmonary dysplasia (BPD) was first described by Northway et al. in 1967 as a new lung disease in preterm infants following respiratory distress syndrome [1]. Over the past 50 years since its first characterization, medical technology and clinical management have evolved. Accordingly, the pathology of BPD evolved from primarily necrotic bronchiolitis and fibrotic changes in the lung tissues to alveolar simplification in the post-surfactant era [1, 2]. BPD is associated with long-term cardiopulmonary complications as well as neurodevelopmental disadvantages, including cerebral palsy, vision and hearing deficits, and mental and psychomotor impairments [3–12].

Due to how it is defined clinically, the diagnosis of BPD remains rather subjective. Most contemporary practices follow the 2001 NICHD Workshop definition to diagnose BPD at 36 weeks postmenstrual age (PMA) [13]. In this version, severity stratification (mild, moderate, or severe) was based on the duration of supplemental oxygen and respiratory support use. Diagnostic criteria were revised after the 2016 NICHD Workshop [14]. In 2019, a study that took an evidence-based approach by correlating 18 potential definitions of BPD with toddler-age respiratory and neurodevelopmental outcomes found that the best way to define BPD was the use of the respiratory support mode at 36 weeks of PMA. [15]. In this version, a grading system (Grade 1, 2, or 3) was introduced.

It has been shown that supplemental oxygen use in the first two weeks of life correlates with BPD severity [16]. Subsequently, a BPD outcome estimator using demographic and respiratory factors, including supplemental oxygen and respiratory support mode, was developed [17]. The goal of the estimator was to calculate BPD probabilities for each severity level. Recently, the estimator was revised to accommodate the BPD grading system [18]. The fair performance of the revised prediction model suggests more studies may be needed to identify additional early-life predictors. The objective of this study was to investigate whether oxygenation index (OI) dynamics in the first three weeks of life correlate with BPD grade.

## Methods

### Study participants

Preterm infants born between 2018 and 2020 with a birth gestation between 23 weeks 0 days and 29 weeks 6 days admitted to the neonatal intensive care unit (NICU) at the Loma Linda University Children’s Hospital (LLUCH) or the Riverside University Health System (RUHS) were assessed for inclusion. Infants born before 2018 were not included because the Tiny Baby Program was established in 2018. The program provided protocolized respiratory management for all infants born at less than 30 weeks of gestation [19]. The study was approved by local Institutional Review Boards (LLUCH IRB#: 5200338; RUHS IRB#: 1689889) with a waiver of informed consent due to the retrospective nature of the study. Only infants who survived the NICU course and had arterial blood gas (ABG) data in the first three weeks of life were included. In this practice, arterial access (either through the umbilical artery or the radial artery) is routinely established for infants born less than 30 weeks’ gestation requiring respiratory support upon NICU admission. Infants transferred to another facility before 36 weeks postmenstrual age (PMA) were excluded due to the unavailability of respiratory support data for BPD grade assignment. Infants who were sent home on room air before 36 weeks of PMA were classified as having no BPD. These infants were included in the analysis.

### Data extraction and curation

ABG, respiratory support, as well as demographic and medication data, were retrieved from the clinical medical records database by a hospital data architect (A.N.), followed by manual curation by one research team member (R.M.L.), including entering and verifying mean airway pressure (MAP) and the fraction of inspired oxygen (FiO_2_) around the time when an ABG was obtained. For continuous positive airway pressure (CPAP), the positive end expiratory pressure (PEEP) was considered MAP. The oxygenation index (OI) was calculated by dividing the product of FiO_2_ and MAP by the partial pressure of oxygen (PaO_2_).

### Definition of variables

Birth weight (BW) was converted to a birth weight z-score (BW-Z) using the 2013 Fenton sex-specific growth charts [20]. Gestational age (GA) in completed weeks was included in the multinomial regression analysis. Each blood gas report had an associated timestamp, which was used to calculate the day of life (DOL). One DOL was defined as a 24-h interval beginning at birth. Decimal points were allowed when calculating DOL in trajectory modeling. DOL as an integer was used for the multinomial regression analysis. Dexamethasone administration data was obtained from the medication administration records. Infants were considered to have received dexamethasone if the medication was given for 10 days or more. The average daily OI change rate (DOI^AVG^) for each infant was calculated as follows: Step 1—calculate the mean OI for the first and last DOLs for which OI data was available; Step 2—calculate the difference between the first and last mean OI values; Step 3—divide the difference in mean OI by the total number of DOLs (full days) between the two mean OI values. Those infants with only one mean OI value were excluded, as it was impossible to calculate DOI^AVG^. BPD grade was determined at 36 weeks PMA based on respiratory support following the publication by Jensen et al. in 2019 [15]. In brief, nasal cannula support of ≤ 2 L per minute (LPM) was classified as Grade 1, nasal cannula support of > 2 LPM or non-invasive positive pressure support was classified as Grade 2, and invasive positive pressure support is classified as Grade 3. Throughout this report, Grade 1 was considered Low-Grade, whereas Grades 2 and 3 were considered High-Grade.

### Statistical analysis and trajectory modeling

For inferential statistics, chi-squared tests were used for categorical variables. One-way analysis of variance was used for continuous variables. All analyses were performed in R version 4.1.1 [21].

We used generalized additive modeling (GAM) and generalized additive mixed modeling (GAMM) techniques for OI trajectory modeling. GAMM is an extension of GAM, which builds models based on piecewise linear functions followed by penalties for wiggliness. Random-effects in the GAMM models take into consideration heterogeneity in individual infants’ trajectories to capture the temporal relationship of repeated measurements within each infant. Both random intercepts and random slopes were allowed in the GAMM models. We first fitted a GAMM model using a single smooth function of Day of Life (DOL) with data points from all infants combined (Model 1). To assess the role of mixed-effects on the modeled OI trajectory, a GAM model was fitted for comparison (Model 2). Subsequently, we fitted 3 GAMM models to assess differential OI trajectories in different BPD categories (No BPD, Low-Grade BPD, and High-Grade BPD): the first model had the BPD categorical variable included as the main-effect (Model 3); the second model had BPD included as a DOL-by-BPD interaction term (Model 4); and the third model had BPD included as both the main-effect and a DOL-by-BPD interaction term (Model 5). The smooth level and parameters were determined by maximum likelihood when models were developed for goodness-of-fit comparison and by restricted estimation of maximum likelihood (REML) for the final model. Akaike information criteria (AIC) were used for model comparison among Models 3–5. For sensitivity analysis, a subset of infants with average OI data points in more than one DOL (*n* = 242) were used. The *mgcv* and *gamm4* packages for R were used for trajectory modeling [22, 23]. Trajectory estimates and standard errors were presented. The pairwise comparison of differences in trajectory estimates was performed following a published method [24].

The frequency of OI data points was based on clinical indications of ABG and the availability of arterial access. Not all infants had OI data points on each day of the first three weeks of life. It was known that likelihood-based methods (e.g., GAMM) provided unbiased estimates when data were subject to missing at random (MAR; e.g., lack of arterial blood access). On the other hand, the trajectory estimates could be biased when data were missing not at random (MNAR; e.g., improved clinical condition negating the need for ABG after a certain DOL or death). Therefore, we conducted sensitivity analyses to study the best- and worst-case scenarios for comparison: for the worst-case scenario, we included only infants with OI data points at the end of the first three weeks of life for modeling, as these infants were assumed to be the most ill clinically; to create the best-case scenario, we imputed missing values by the lowest available OI among all observed for each infant when the OI data points were truncated after a certain DOL.

The association between initial OI or DOI^AVG^ and BPD grade was assessed using multinomial regression analysis, in which BPD grade was considered the dependent outcome variable. Initial OI values and DOI^AVG^ were both included as independent variables in the model. Additionally, GA, BW-Z, sex, and the duration of invasive ventilation within the first three weeks of life (Duration^INV^) were included as covariates for model comparison, for which AIC scores were used. The odds ratio and 95% confidence interval (CI) were presented. The *nnet* package for R was used for the multinomial regression analysis [25].

## Results

**Fig. 1:**
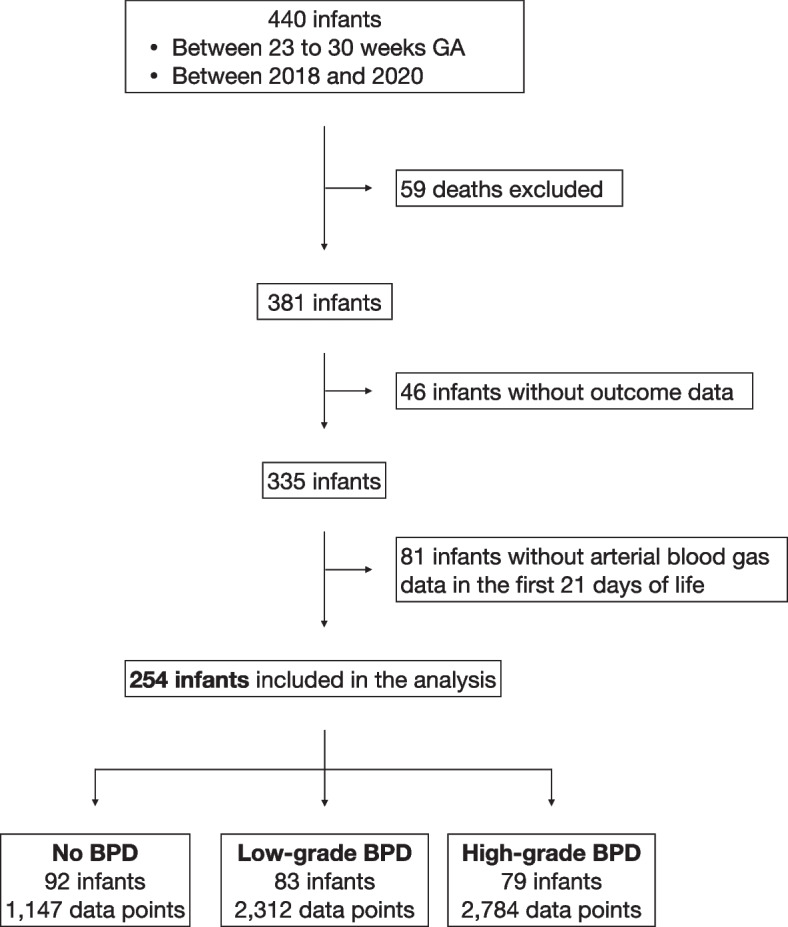
A flowchart depicting the infant inclusion and exclusion processes and the final number of infants in each subgroup for the analysis

A total of 440 infants met GA and birth year criteria for further assessment of inclusion eligibility. Among the 59 infants who died, 36 had OI data. Their median (interquartile range) age of death for these infants was 8 (2–16) days. The cause of death is available in Supplemental Table 1. After excluding infants who died, among the 381 surviving infants, 335 had sufficient respiratory data for BPD grading, and 254 had at least one ABG analysis done in the first three weeks of life for trajectory modeling (Fig. 1). A demographic summary of these 254 infants is available in Table 1. As expected, infants without BPD were born at a later GA, had a higher BW, and were more likely to be female. Dexamethasone was more likely to be administered to infants with High-Grade BPD. Among infants with BPD, those who received the first dose of dexamethasone before DOL 21 were more likely to be in the Low-Grade BPD subgroup (*p*-value = 0.013).

**Table 1:** Infant characteristics

	**All**	**No BPD**	**BPD**		***P*-value**
			**Low-Grade (Grade 1)**	**High-Grade (Grade 2/3)**	
**Number of infants**	254	92	83	79	
**Number of OI data points**	6,243	1,147	2,312	2,784	
**Birth gestation (week)**	26.7 ± 1.8	27.8 ± 1.3	26.1 ± 1.8	26.0 ± 1.7	< 0.001^*^
**Birth weight (gram)**	915 ± 287	1,108 ± 255	846 ± 254	761 ± 224	< 0.001^*^
**Female, n (%)**	119 (47%)	53 (58%)	39 (47%)	27 (34%)	0.009^*^
**Dexamethasone for BPD, n (%)**	52 (20%)	0 (0%)	15 (18%)	37 (47%)	< 0.001^*^
* Started before DOL 21*	11 (21%)	-	7 (47%)	4 (11%)	0.013^†^
**Average daily OI change in the first 21 DOLs**	-0.06 ± 0.68	-0.33 ± 0.52 (*n* = 85)	-0.04 ± 0.75 (*n* = 82)	0.22 ± 0.65 (*n* = 75)	< 0.001^*^

*BPD* Bronchopulmonary dysplasia, *DOL* Day of life (24-h interval), *OI* Oxygenation index

^*^Three-group comparison among No BPD, Low-Grade BPD, and High-Grade BPD

^†^Chi-squared test comparing infants who received dexamethasone starting before vs. after DOL 21 between Low- and High-Grade BPD subgroups

**Fig. 2:**
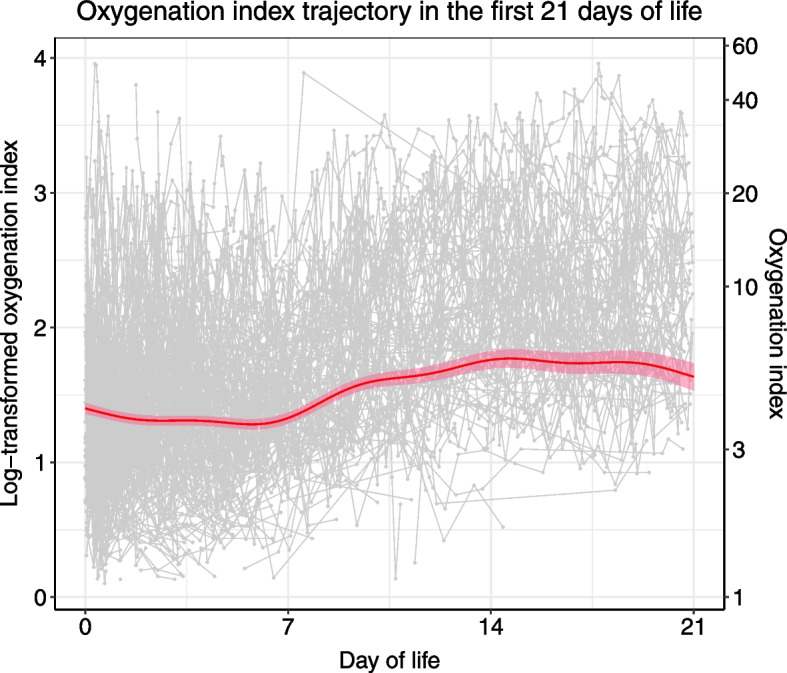
Oxygenation index trajectory estimates over the first three weeks of life after logarithmic transformation using generalized additive mixed modeling. The red line represents the trajectory estimates. The shade represents the standard error of the trajectory estimates. The gray dots represent the raw OI values at the indicated time points. The raw OI values for each infant were connected by a line. The log scale is shown on the left Y-axis, and the linear scale is shown on the right

The distribution of OI for each time point was skewed; therefore a logarithmic (log) transformation was performed prior to trajectory modeling (Supplemental Fig. 1). Using all OI data points for modeling, irrespective of BPD grade subgroups, the OI trajectory estimates stayed around 5 over the first 3 weeks of life (Fig. 2). To contrast the difference between models with and without mixed-effects, we also modeled OI with GAM, in which all data points were treated as independent from each other. The result showed an upward skew as the DOL increased (Supplemental Fig. 2).

**Fig. 3:**
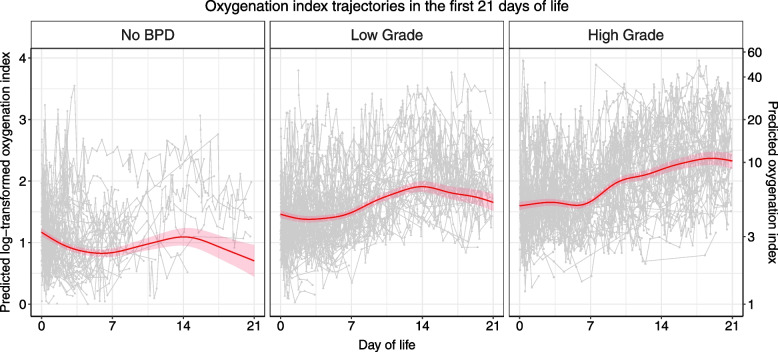
Oxygenation index trajectory estimates by BPD severity over the first three weeks of life after logarithmic transformation using generalized additive mixed modeling. Low-Grade BPD represents Grade 1 BPD, whereas High-Grade BPD represents Grade 2/3 BPD based on the criteria proposed by Jensen et al. [12]. The red lines represent trajectory estimates. The shade represents the standard error of the trajectory estimates. The gray dots represent the raw OI values at the indicated time points. The raw OI values for each individual infant were connected by a line. The log scale is shown on the left Y-axis, and the linear scale on the right

We then modeled OI trajectories for each BPD grade subgroup by introducing the variable as a main-effect term and/or as an interaction term for the smooth function (Models #3–5; see Methods for details). AIC (Model #3 AIC: 7145; Model #4 AIC: 7215; Model #5 AIC: 7119) selected the model with the BPD grade variable both as a main-effect term and an interaction term (Model #5). The expected values of OI on DOL 0 were the lowest in the No-BPD group (Fig. 3). Furthermore, the trajectory in this group showed a downward trend, as opposed to a flat and an upward trend in the Low-Grade and High-Grade BPD subgroups, respectively (Fig. 3).

**Fig. 4:**
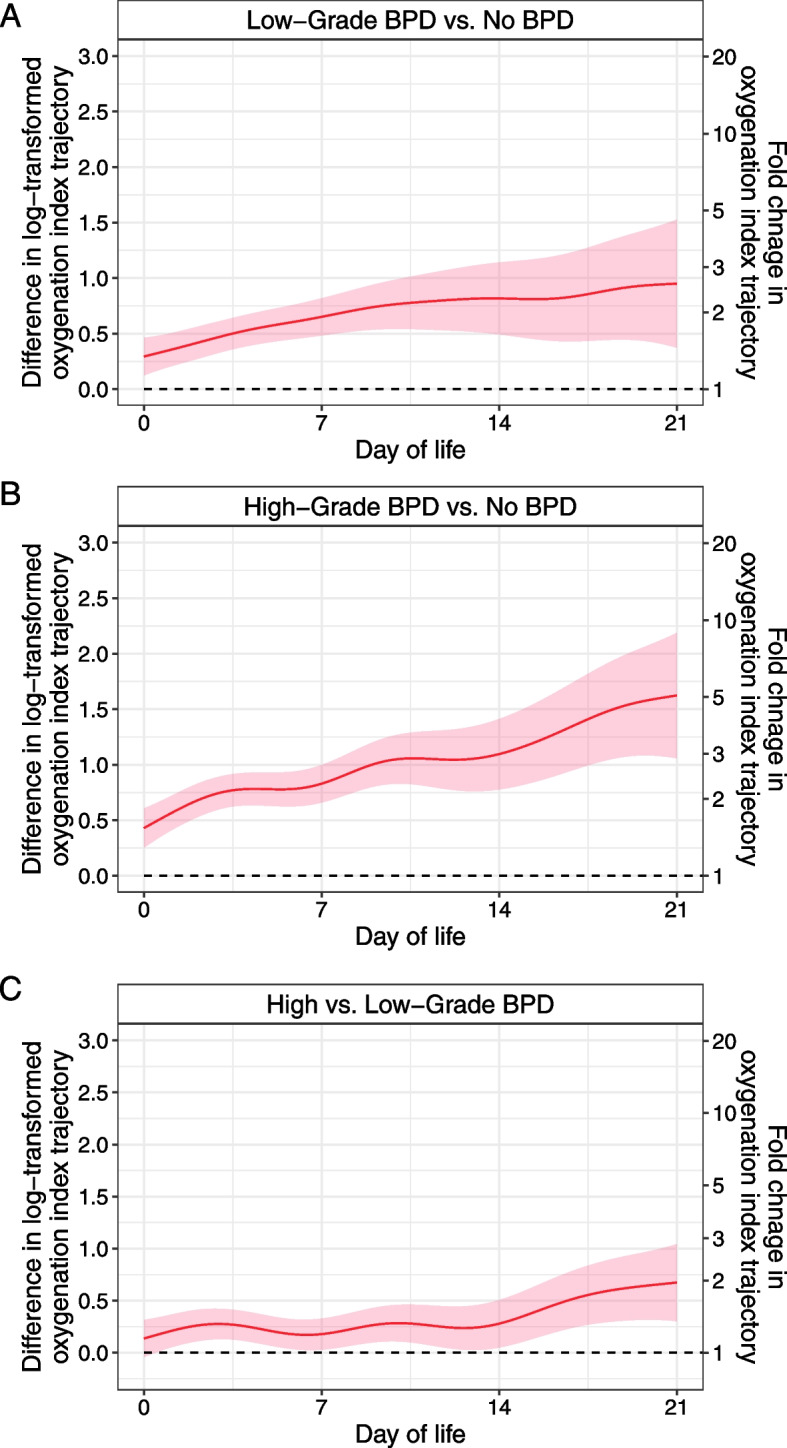
Pairwise differences between the modeled log-transformed oxygenation index trajectory curves. Red lines represent expected differences in trajectory curves between the indicated BPD groups. The shade represents a 95% confidence interval. The log scale is shown on the left Y-axis, and the linear scale of the corresponding fold difference is shown on the right

We then performed pairwise comparisons of log-transformed OI trajectories among the three BPD grade subgroups (Fig. 4) [24]. Notably, log-transformed OI differences correspond to fold changes in OI as a result of log transformation. The differences between log-transformed OI trajectories in both Low-Grade and High-Grade BPD subgroups when compared to the No-BPD subgroup showed a steady increase over time, with wider differences between the High-Grade BPD and No-BPD subgroups. The difference in log-transformed OI trajectory estimates between the Low-Grade and the High-Grade BPD subgroups was around 0.25 (corresponding to a 1.3-fold difference in OI) between DOL 0 and 14. After DOL 14, the difference gradually increased to approximately 0.75 (a near twofold increase in OI) on DOL 21.

Out of the 254 infants with OI data, 12 infants only had data points within one DOL, which may potentially lead to unreliable trajectory estimates. To test this, we excluded data points from these 12 infants and repeated trajectory modeling in a sensitivity analysis. Trajectory estimates for each BPD category remained nearly identical (Supplemental Fig. 3). To further address the uncertainty of trajectory curves in the presence of missing values (see Methods for details), additional models for the best- and worst-case scenarios were developed for comparison (Supplemental Fig. 4).

**Fig. 5:**
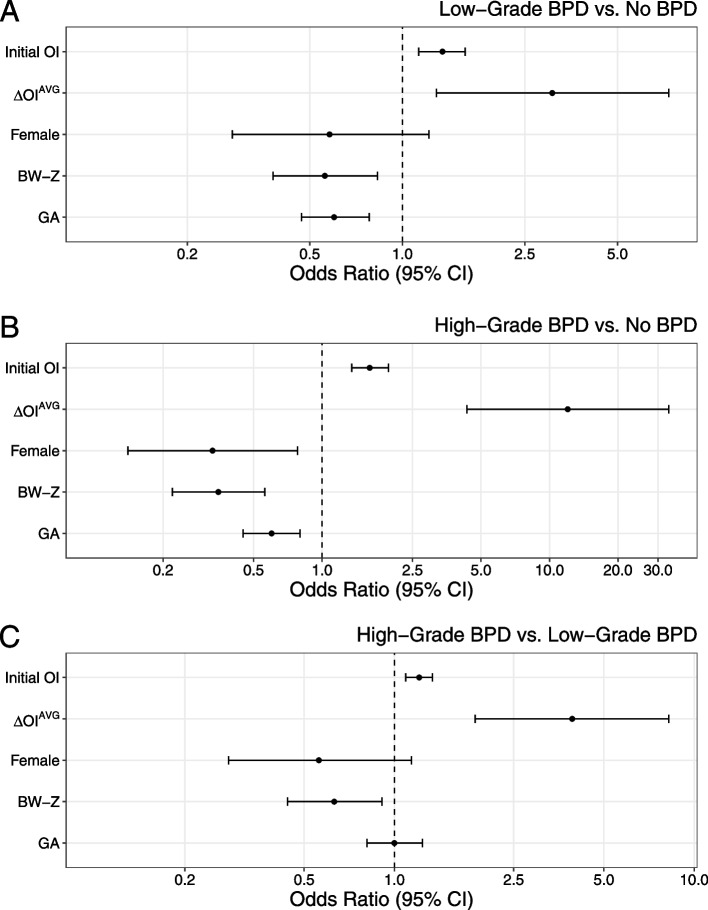
Multinomial regression analysis of BPD severity. Forest plots showing the odds ratio value and 95% confidence interval (CI) for each of the indicated variables after adjusting for the others

Based on the findings from the GAMM models, we further hypothesized that changes in OI in the first three weeks of life were associated with BPD grade at 36 weeks PMA. To test this, we calculated ΔOI^AVG^ from the 242 infants with OI data points in more than one DOL. Multinomial regression models were then developed to assess the association between OI dynamics (initial OI and ΔOI^AVG^) and BPD grade subgroups. Aside from initial OI and ΔOI^AVG^, four other well-characterized risk factors for BPD, namely birth GA, BW-Z, sex, and Duration^INV^, were also assessed for inclusion in the model [26]. Various combinations of the covariates were compared (Supplemental Table 2) using AIC. The model that included the initial OI, ΔOI^AVG^, GA, BW-Z, and sex had the lowest AIC and was selected as the final model (Model #32). In this model, we found that an increase in ΔOI^AVG^ by 1 was independently associated with a threefold increase in the odds of having Low-Grade BPD when compared to No BPD, with an OR of 3.07 (95% CI: 1.29–7.34), as well as a fourfold increase in the odds of having High-Grade BPD in comparison to Low-Grade BPD (OR: 3.92, 95% CI: 1.86–8.23) (Fig. 5). The initial OI values were also independently associated with increased odds of developing Low-Grade and High-Grade BPD (Low-Grade vs. No BPD: OR 1.35, 95% CI: 1.13–1.6; High-Grade vs. No BPD: OR 1.62, 95% CI: 1.35–1.96), as well as increased odds of High-Grade BPD when compared to Low-Grade BPD (OR: 1.21, 95% CI: 1.09–1.34) (Fig. 5). Aside from the initial OI and ΔOI^AVG^, BW-Z was the only variable that showed a grade-dependent association with the BPD grade outcomes (Fig. 5). Interestingly, while Duration^INV^ in the first three weeks of life was significantly associated with a higher BPD grade after adjusting for GA, BW-Z, and sex (Supplemental Fig. 5), the association became non-significant after adjusting for the initial OI and ΔOI^AVG^ (Supplemental Fig. 6). AIC also did not favor a model that included Duration^INV^ (Model #36 in Supplemental Table 2) compared to the model without Duration^INV^ (Model #32 in Supplemental Table 2).

## Discussion

In this study, we performed OI trajectory analysis followed by regression analysis using OI data in the first three weeks of life and identified OI dynamics as a predictor for BPD grade in preterm infants born before 30 weeks of gestation.

OI is used to characterize the severity of pulmonary illnesses. OI is calculated by taking the ratio between the degree of respiratory support and blood oxygen content. The higher the OI, the more severe the pulmonary condition is. OI has not been routinely used to describe the severity of respiratory distress syndrome in newborns, a disease of pulmonary immaturity, although it is commonly used in neonatal intensive care to assess the need for support escalation to extracorporeal membrane oxygenation. The OI trajectories for the No BPD, Low-Grade BPD, and High-Grade BPD subgroups provided a way to visually inspect the bulk movement of the OI values over the first three weeks of life. The modeled trajectory curves were not adjusted for perinatal or demographic factors and were more hypothesis-generating than hypothesis-testing. An infant supported on CPAP of 5 cmH_2_O at 30% FiO_2_ with a PaO_2_ of 50 mmHg has an OI of 3; an infant supported on CPAP of 6 cmH_2_O at 30% FiO_2_ with a PaO_2_ of 45 mmHg has an OI of 4; and an infant supported on CPAP of 6 cmH_2_O at 40% FiO_2_ with a PaO_2_ of 50 mmHg has an OI of 4.8. These examples are representative of the respective expected starting points of respiratory support in the three BPD grade subgroups based on the modeled curves, although the variations were wide. The infants in the No BPD subgroup had stable OI in the first two weeks of life, followed by a downward trend during the third week of life. On the other hand, infants who were diagnosed with BPD (Low-Grade or High-Grade) had an increase in OI in the first two weeks of life. However, those with High-Grade BPD continued to have an uptrending trajectory during the third week of life, while those with Low-Grade BPD showed a steady or downtrending OI trajectory. These findings suggest that the degree of respiratory support during the third week of life may be key to predicting High vs. Low Grade BPD. Interestingly, these findings coincided with the percentage of infants who received dexamethasone before DOL 21. It is possible that dexamethasone may have effectively improved respiratory status, leading to a decrease in OI, and subsequently affected BPD grade, although this project was not specifically designed to answer the question regarding the association between dexamethasone and BPD grade changes. Nonetheless, it may be desirable to introduce non-pharmacological or pharmacological measures during the third week of life to decrease OI and the risks of High-Grade BPD.

Based on the findings from the analysis of the OI trajectories, we developed regression models to quantify the contribution of OI dynamics to BPD grade. Covariates including birth GA, BW-Z, sex, and Duration^INV^ were assessed for inclusion in the model using AIC. AIC assesses the quality of the model by balancing maximum likelihood and the complexity of the model, with an attempt to control model overfitting. The selected model contained all variables except for Duration^INV^. Forcing Duration^INV^ into the model did not change the roles of the initial OI and ΔOI^AVG^. Although Duration^INV^ was significantly associated with higher BPD grades even after adjusting for GA, BW-Z, and sex, the strength of the association was mitigated after adjusting for the initial OI and ΔOI^AVG^.

GAMM is a powerful technique for trajectory modeling [27, 28]. We have used the technique to model postnatal weight trajectories and probabilities of various BPD severity categories over time [29, 30]. Although the backbone technique, GAM, does not require a linear assumption between the independent and outcome variables, the technique still allows for linear regression if a linear relationship pattern between the independent and dependent variables does exist. In the presence of a non-linear pattern, overfitting is regulated by the smooth parameter. Mixed-effects modeling by GAMM allows the model to consider clusters of data points from each infant, rather than treating all data points independently as in GAM. Therefore, GAMM is more likely to accurately describe the trajectory. When mixed modeling was not used, we observed an upward skew in the modeled trajectory due to the nature of the dataset, as sicker infants who required higher respiratory support (higher OI) and more frequent blood gas assessments (more data points) predominated the later time points. GAMM tolerates MAR missing values well, even when the missing values cause left or right data censoring. However, the model would become unreliable when MNAR is the only type of missing value, such as in the case where ABG is no longer needed due to improved clinical status or death. Despite proactively excluding those infants that died, the missing value issue in our study still existed and likely resulted from a mixture of both MAR and MNAR. To address the uncertainty, we created the best- and worst-case scenarios using a subset of data points as well as missing value imputations to provide an upper and lower boundary within which the “true” trajectory might fall. Nonetheless, not having OI data for the entire duration was a major limitation of our study.

Being a retrospective and single-center study was another limitation. Given the retrospective nature of the study, we were only able to assess average daily changes in OI from the available data. OI measurement requires arterial access to obtain ABG. Due to the risks involved, arterial access may not be performed routinely in mildly to moderately ill newborns. Studies have assessed the efficacy of using modified versions of OI, such as the oxygenation saturation index (OSI, which replaces PaO_2_ with blood oxygen saturation using pulse oximetry, or SpO_2_, in index calculation) or respiratory severity scores (RSS, the product of MAP and FiO_2_), as surrogate markers for pulmonary illness [31–37]. One recent study showed a strong correlation between OI and OSI in the neonatal population [35]. Another study showed a strong correlation between OI and RSS [31].

One of the most significant advantages of the BPD grading system is its evidence-based approach to correlating the BPD grade with respiratory and neurodevelopmental outcomes during its development. Grade 3 BPD had significantly worse respiratory outcomes and morbidities of prematurity [38]. The NICHD BPD outcome estimator was recently updated to accommodate BPD grade prediction [18]. However, the model only provides fair performance, suggesting that there is a need to search for additional predictors. Identifying factors that may be used to predict High-Grade BPD may allow for early targeted interventions and an opportunity to at least “downgrade” BPD if not completely prevent its occurrence. A short-term improvement in BPD grade may translate into long-term neurodevelopmental and cardiopulmonary benefits. As arterial catheter placement may not be feasible or indicated in all at-risk infants, future investigation may focus on assessing whether OSI or RSS may serve as useful alternatives to OI as predictors when validating our findings.

In conclusion, we characterized OI trajectories in a retrospective cohort and quantified the association between OI and BPD grades. These findings suggest that adding OI dynamics to the list of predictors for BPD grades may be beneficial and may provide a path towards a more accurate early identification of those at risk for the high-grade disease.

## Supplementary Information

**Additional file 1: Supplemental Figure 1.** Boxplots depicting the distribution of oxygenation index data points for each day of life on (A) a linear or (B) a logarithmic (log) scale. The thick horizontal lines represent median, the upper and lower hinges of the boxes represent the 75^th^ and 25^th^ percentiles, and the whiskers extend from the hinges to the largest value no further than 1.5 times the interquartile range. Dots represent outliers. **Supplemental Figure 2.** Oxygenation index trajectory estimates over the first three weeks of life after logarithmic transformation using generalized additive mixed modeling (solid line) or generalized additive modeling (dotted line). The red lines represent trajectory estimates. The shades represent the standard error of the trajectory estimates. The grey dots represent the raw OI values at the indicated time points. The raw OI values for each individual infant were connected by a line. The logarithmic scale is shown on the left Y-axis, and the linear scale on the right. **Supplemental Figure 3.** Oxygenation index (OI) trajectory estimates over the first three weeks of life after logarithmic transformation using generalized additive mixed modeling. The input data contained either all infants with at least one OI data point (red, n=254), or infants with average OI values in more than one DOL/24-hr interval (blue, n=242). The corresponding shades represent standard error of the trajectory estimates. The logarithmic scale is shown on the left Y-axis, and the linear scale on the right. Not that the two trajectory curves are nearly identical, indicating that including or excluding infants contributing data points only within one DOL/24-hr interval does not significantly alter trajectory estimates. **Supplemental Figure 4.** Oxygenation index (OI) trajectory estimates over the first three weeks of life after logarithmic transformation using generalized additive mixed modeling (GAMM) with and without missing value imputation. Red curves represent trajectory estimates using all available OI data points based on the assumption that the missing OI values were missing at random. Blue solid curves are trajectory estimates modeled using data from a subset of infants who had OI data points at the end of the three weeks of life, representing the worst-case scenario. Blue dashed lines are trajectory estimates modeled using all available OI data points plus imputed data by taking the lowest observed OI value for each infant, representing the best-case scenario in which arterial blood gas was no longer required as a result of stable respiratory status (missing not at random). Shades represent standard error of the trajectory estimates. **Supplemental Figure 5.** Multinomial multivariable regression analysis of BPD grades. Forest plots showing odds ratio and 95% confidence interval (CI) for each of the indicated variables after adjusting for the others. Note that the duration of invasive ventilation (Duration^INV^) in the first three weeks of life significantly correlated with BPD grade in each of the pairwise comparison after adjusting for gestational age (GA), birth weight z-score (BW-Z), and sex. **Supplemental Figure 6.** Multinomial multivariable regression analysis of BPD grade. Forest plots showing odds ratio (OI) and 95% confidence interval (CI) for each of the indicated variables after adjusting for the others. Note that the odds ratios for the initial OI and the average daily OI change (ΔOI^AVG^) remained significant after adjusting for gestational age (GA), brith weight z-score (BW-A), and sex with and without additional adjusting by the duration of invasive ventilation (Duration^INV^). GA. **Supplemental Table 1.** Cause of death. **Supplemental Table 2.** Multinomial regression model comparison using Akaike information criteria (AIC).

## Data Availability

The de-identified datasets used and analyzed during the current study are available from the corresponding author on reasonable request.

## Abbreviations

AIC: Akaike information criteria
BPD: Bronchopulmonary dysplasia
BW-Z: Birth weight z-score
CI: Confidence interval
CPAP: Continuous positive airway pressure
DOI^AVG^: Average daily oxygenation index change
DOL: Day of life
Duration^INV^: Duration of invasive ventilation
FiO_2_: Fraction of inspired oxygen
GA: Gestational age
GAM: Generalized additive modeling
GAMM: Generalized additive mixed modeling
IRB: Institutional Review Board
LLUCH: Loma Linda University Children’s Hospital
LPM: Liter per minute
MAP: Mean airway pressure
MAR: Missing at random
MNAR: Missing not at random
NICHD: Eunice Kennedy Shriver National Institute of Child Health and Human Development
NICU: Neonatal intensive care unit
OI: Oxygenation index
OR: Odds ratio
OSI: Oxygenation saturation index
PaO_2_: Partial pressure of oxygen
PEEP: Positive end expiratory pressure
PMA: Postmenstrual age
REML: Restricted estimation of maximum likelihood
RUHS: Riverside University Health System
RSS: Respiratory severity score
