# Supplementary material for: Oxygenation index in the first three weeks of life is a predictor of bronchopulmonary dysplasia grade in very preterm infants
Source: BMC Pediatr. 2023 Jan 13;23:18. doi: 10.1186/s12887-023-03835-3 (PMC9838074; doi:10.1186/s12887-023-03835-3)
Supplement: Supplementary file 1 — Additional file 1: Supplemental Figure 1. Boxplots depicting the distribution of oxygenation index data points for each day of life on (A) a linear or (B) a logarithmic (log) scale. The thick horizontal lines represent median, the upper and lower hinges of the boxes represent the 75th and 25th percentiles, and the whiskers extend from the hinges to the largest value no further than 1.5 times the interquartile range. Dots represent outliers. Supplemental Figure 2. Oxygenation index trajectory estimates over the first three weeks of life after logarithmic transformation using generalized additive mixed modeling (solid line) or generalized additive modeling (dotted line). The red lines represent trajectory estimates. The shades represent the standard error of the trajectory estimates. The grey dots represent the raw OI values at the indicated time points. The raw OI values for each individual infant were connected by a line. The logarithmic scale is shown on the left Y-axis, and the linear scale on the right. Supplemental Figure 3. Oxygenation index (OI) trajectory estimates over the first three weeks of life after logarithmic transformation using generalized additive mixed modeling. The input data contained either all infants with at least one OI data point (red, n=254), or infants with average OI values in more than one DOL/24-hr interval (blue, n=242). The corresponding shades represent standard error of the trajectory estimates. The logarithmic scale is shown on the left Y-axis, and the linear scale on the right. Not that the two trajectory curves are nearly identical, indicating that including or excluding infants contributing data points only within one DOL/24-hr interval does not significantly alter trajectory estimates. Supplemental Figure 4. Oxygenation index (OI) trajectory estimates over the first three weeks of life after logarithmic transformation using generalized additive mixed modeling (GAMM) with and without missing value imputation. Red curves represe [file 12887_2023_3835_MOESM1_ESM.pdf]

Supplemental Figure 1. Boxplots depicting the distribution of oxygenation index data points for each day of life on (A) a linear or (B) a logarithmic (log) scale. The thick horizontal lines represent median, the upper and lower hinges of the boxes represent the 75th and 25th percentiles, and the whiskers extend from the hinges to the largest value no further than 1.5 times the interquartile range. Dots represent outliers.

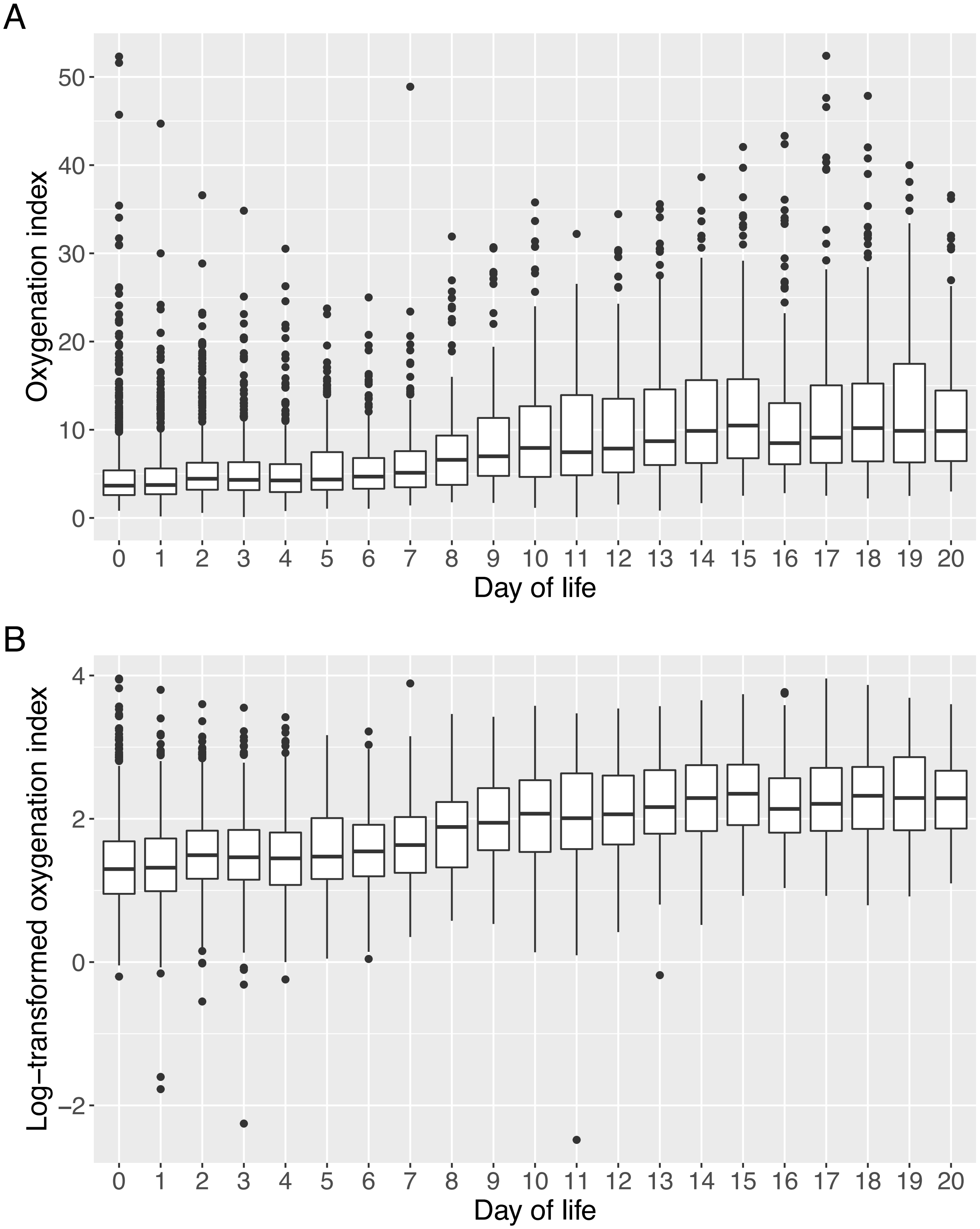

Supplemental Figure 2. Oxygenation index trajectory estimates over the first three weeks of life after logarithmic transformation using generalized additive mixed modeling (solid line) or generalized additive modeling (dotted line). The red lines represent trajectory estimates. The shades represent the standard error of the trajectory estimates. The grey dots represent the raw OI values at the indicated time points. The raw OI values for each individual infant were connected by a line. The logarithmic scale is shown on the left Y-axis, and the linear scale on the right.

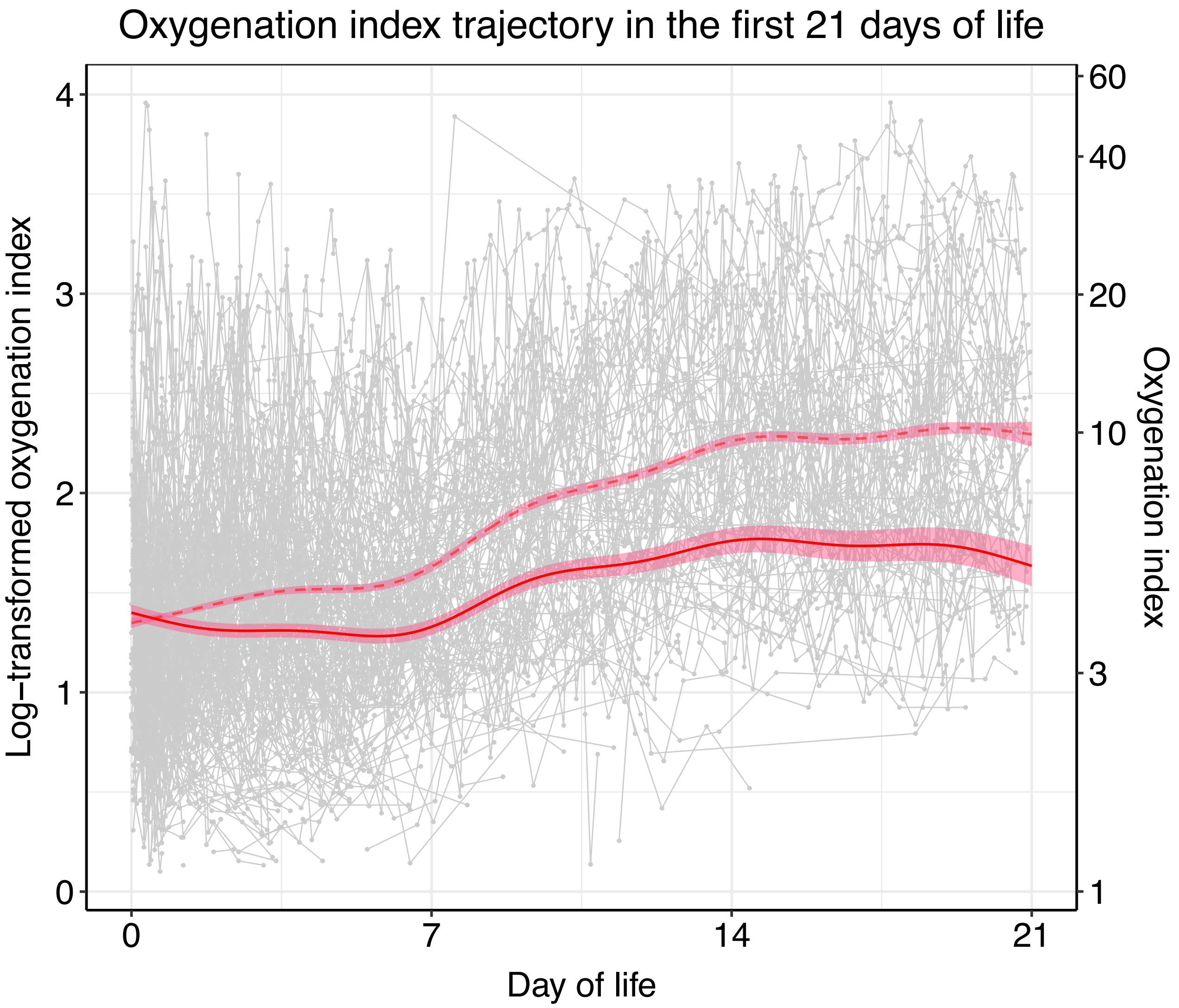

Supplemental Figure 3. Oxygenation index (OI) trajectory estimates over the first three weeks of life after logarithmic transformation using generalized additive mixed modeling. The input data contained either all infants with at least one OI data point (red, n=254), or infants with average OI values in more than one DOL/24-hr interval (blue, n=242). The corresponding shades represent standard error of the trajectory estimates. The logarithmic scale is shown on the left Y-axis, and the linear scale on the right. Not that the two trajectory curves are nearly identical, indicating that including or excluding infants contributing data points within one DOL/24-hr interval does not significantly alter trajectory estimates.

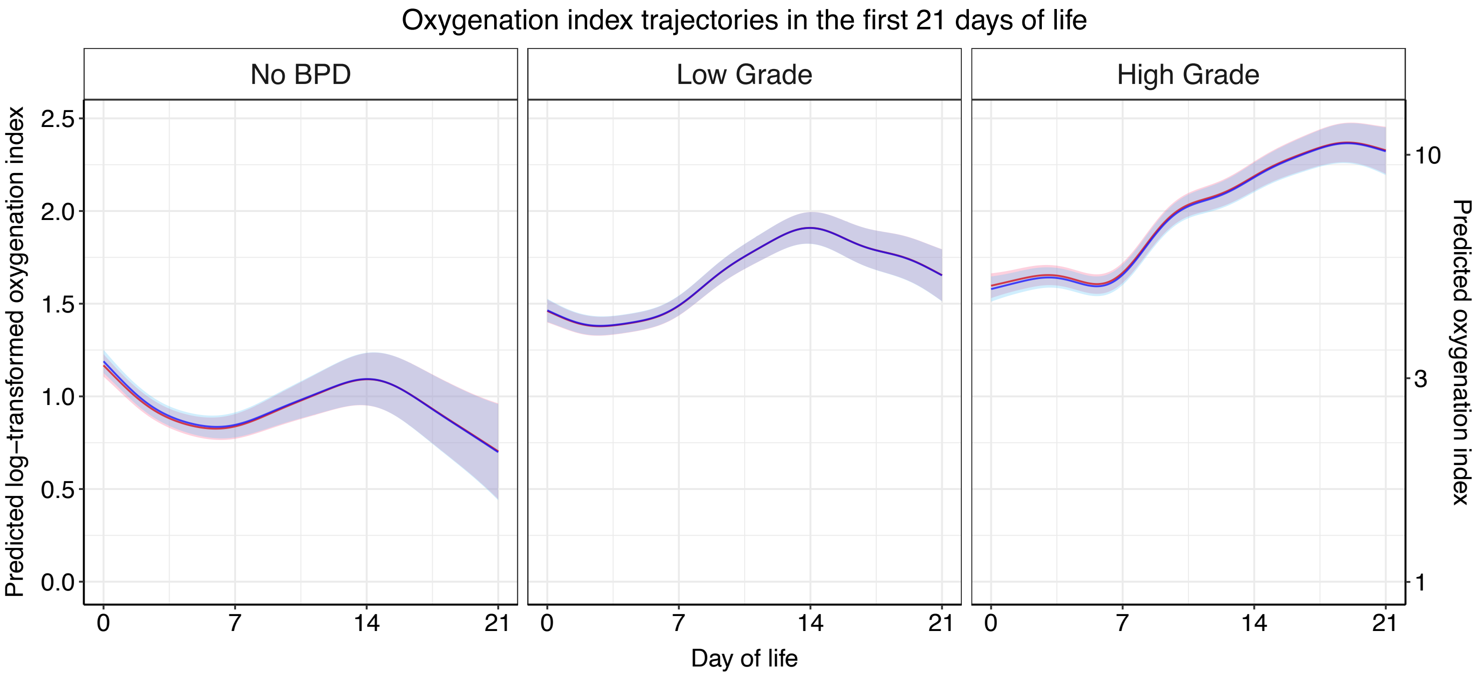

Supplemental Figure 4. Oxygenation index (OI) trajectory estimates over the first three weeks of life after logarithmic transformation using generalized additive mixed modeling (GAMM) with and without missing value imputation. Red curves represent trajectory estimates using all available OI data points based on the assumption that the missing OI values were missing at random. Blue solid curves are trajectory estimates modeled using data from a subset of infants who had OI data points at the end of the three weeks of life, representing the **worst-case** scenario. Blue dashed lines are trajectory estimates modeled using all available OI data points plus imputed data by taking the lowest observed OI value for each infant, representing the **best-case** scenario in which arterial blood gas was no longer required as a result of stable respiratory status (missing not at random). Shades represent standard error of the trajectory estimates.

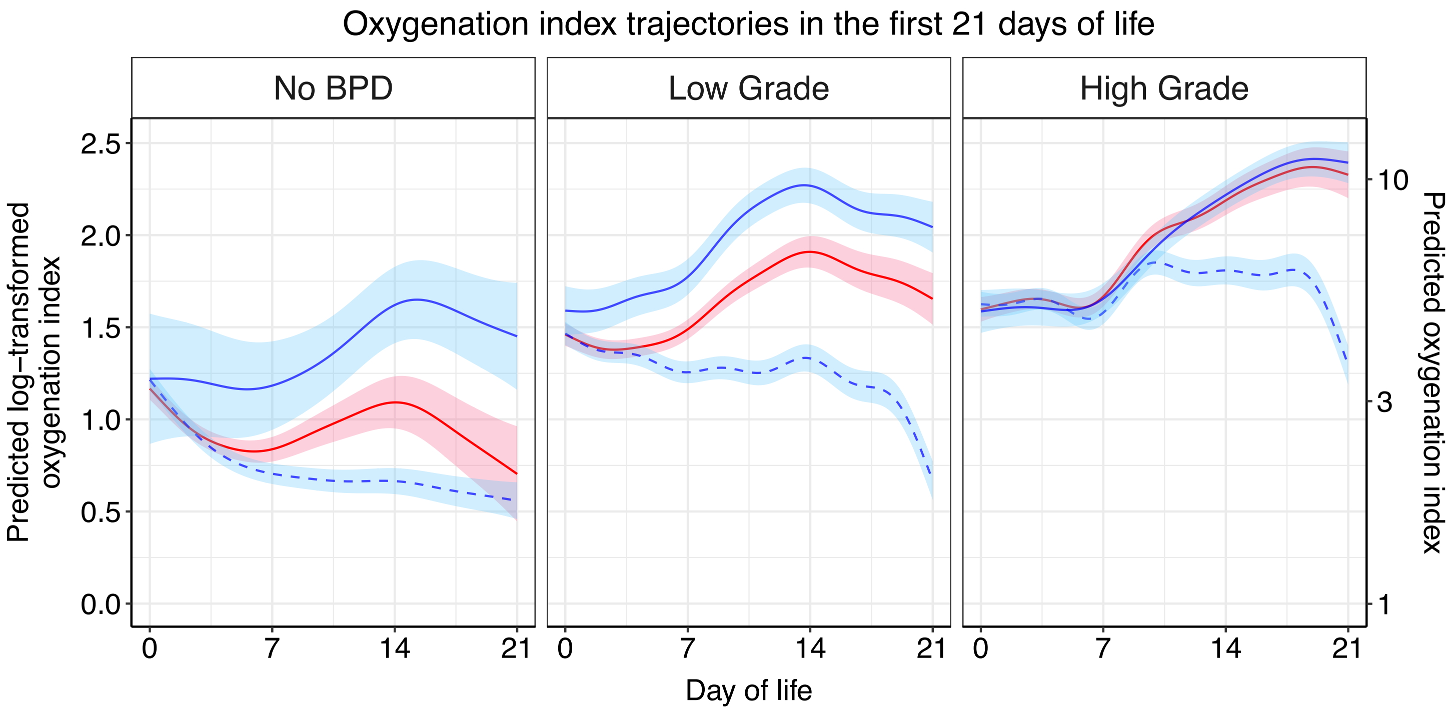

Supplemental Figure 5. Multinomial multivariable regression analysis of BPD grades. Forest plots showing odds ratio and 95% confidence interval (CI) for each of the indicated variables after adjusting for the others. Note that the duration of invasive ventilation ($Duration^{INV}$) in the first three weeks of life significantly correlated with BPD grade in each of the pairwise comparison after adjusting for gestational age (GA), birth weight z-score (BW-Z), and sex.

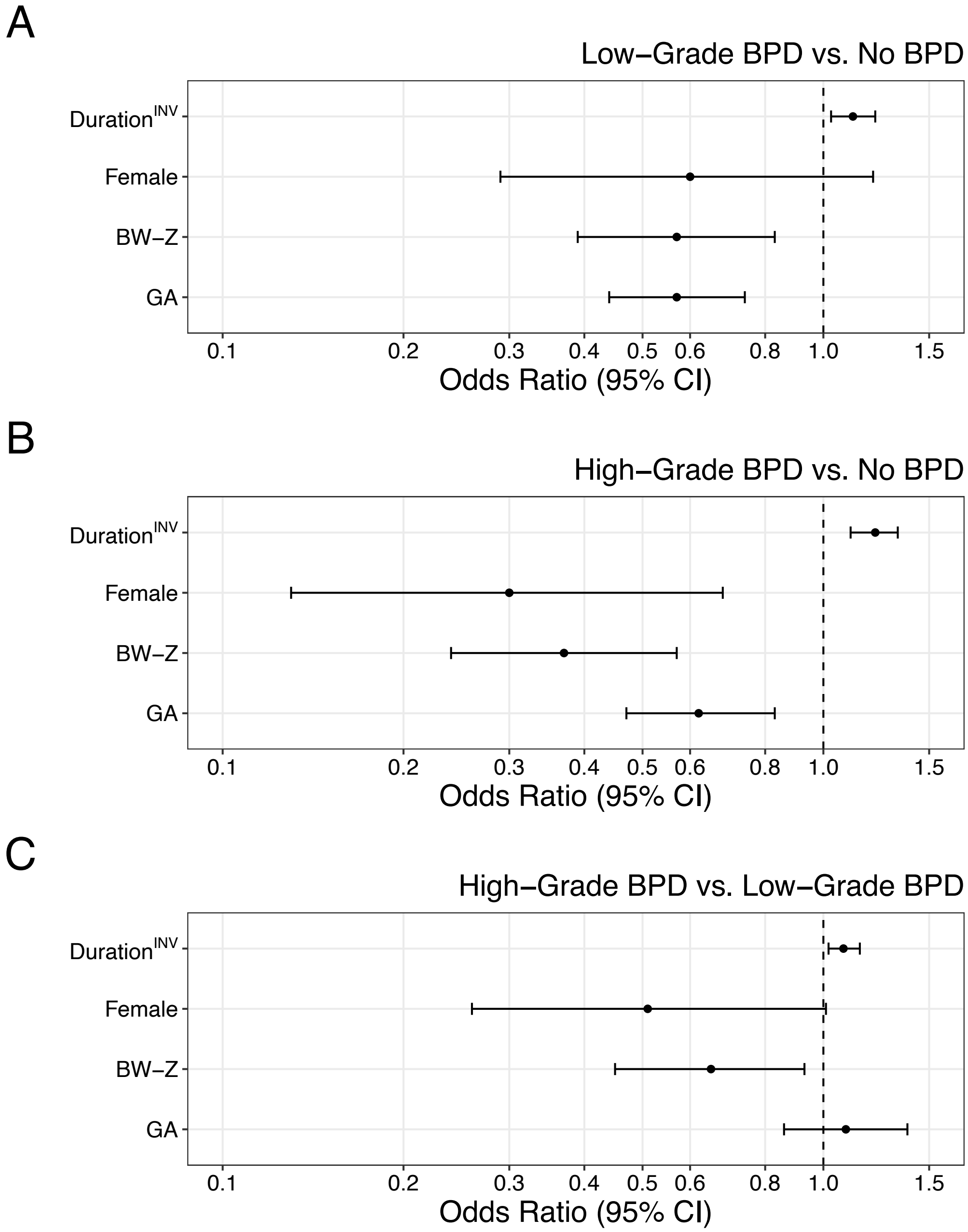

Supplemental Figure 6. Multinomial multivariable regression analysis of BPD grade. Forest plots showing odds ratio (OI) and 95% confidence interval (CI) for each of the indicated variables after adjusting for the others. Note that the odds ratios for the initial OI and the average OI change rate (ΔOI^{AVG}) remained significant after adjusting for gestational age (GA), brith weight z-score (BW-A), and sex with and without additional adjusting by the duration of invasive ventilation ($Duration^{INV}$).

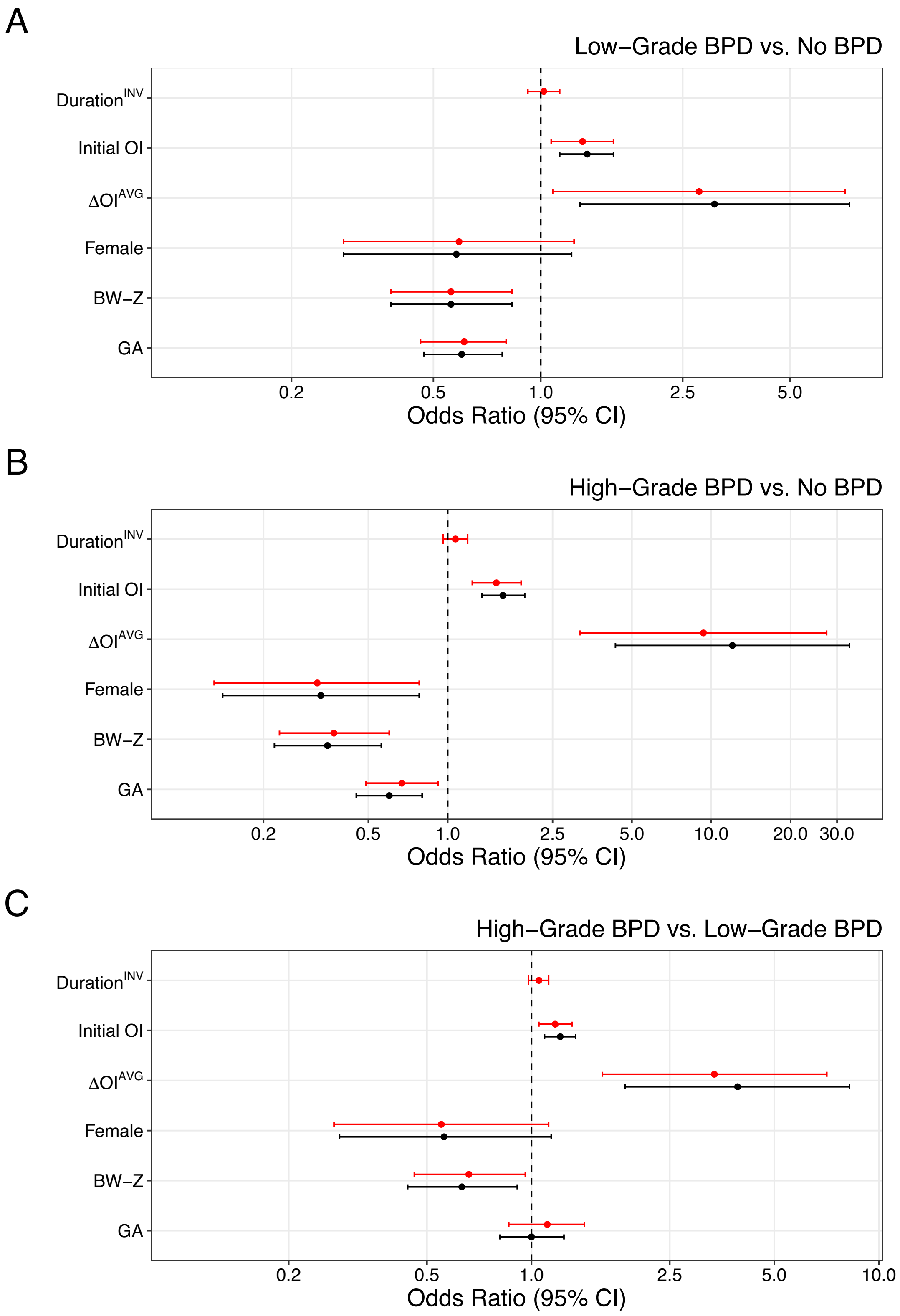

Supplemental Table 1. Cause of death.

Cause of death	Number of infants	Number of infants with OI data
Comfort care	16	9
Congenital anomaly	4	2
Infection	8	7
Massive air leak	6	2
Massive hemorrhage/hypovolemic shock	12	8
NEC/SIP	13	8
**Total**	**59**	**36**

OI: oxygenation index.
NEC/SIP: necrotizing enterocolitis/spontaneous intestinal perforation.

Supplemental Table 2. Multinomial regression model comparison using Akaike information criteria (AIC).

**Model #***	**Independent variable(s) included**	**AIC**
6	GA	481
7	BW-Z	508
8	Sex	531
9	$Duration^{INV}$	470
10	Initial OI, ΔOI^{AVG}	438
11	GA, BW-Z	452
12	GA, Sex	478
13	GA, $Duration^{INV}$	455
14	GA, Initial OI, ΔOI^{AVG}	426
15	BW-Z, Sex	502
16	BW-Z, $Duration^{INV}$	452
17	BW-Z, Initial OI, ΔOI^{AVG}	421
18	Sex, $Duration^{INV}$	464
19	Sex, Initial OI, ΔOI^{AVG}	437
20	$Duration^{INV}$, Initial OI, ΔOI^{AVG}	433
21	GA, BW-Z, Sex	447
22	GA, BW-Z, $Duration^{INV}$	451
23	GA, BW-Z, Initial OI, ΔOI^{AVG}	407
24	GA, Sex, $Duration^{INV}$	451
25	GA, Sex, Initial OI, ΔOI^{AVG}	425
26	GA, $Duration^{INV}$, Initial OI, ΔOI^{AVG}	425
27	BW-Z, Sex, $Duration^{INV}$	446
28	BW-Z, Sex, Initial OI, ΔOI^{AVG}	419
29	BW-Z, $Duration^{INV}$, Initial OI, ΔOI^{AVG}	419
30	Sex, $Duration^{INV}$, Initial OI, ΔOI^{AVG}	432
31	GA, BW-Z, Sex, $Duration^{INV}$	429
**32**	**GA, BW-Z, Sex, Initial OI, ΔOI^{AVG}**	**405**
33	GA, BW-Z, $Duration^{INV}$, Initial OI, ΔOI^{AVG}	409
34	GA, Sex, $Duration^{INV}$, Initial OI, ΔOI^{AVG}	424
35	BW-Z, Sex, $Duration^{INV}$, Initial OI, ΔOI^{AVG}	416
36	GA, BW-Z, Sex, $Duration^{INV}$, Initial OI, ΔOI^{AVG}	406

*Models #1-5 were reserved for GAMM and GAM models, see text.
GA: gestational age at birth, BW-Z: birth weight z-score, DOI^{AVG}: average daily oxygenation index change, $Duration^{INV}$: duration of invasive ventilation.
